# Dead space and CO_2 _elimination related to pattern of inspiratory gas delivery in ARDS patients

**DOI:** 10.1186/cc11232

**Published:** 2012-03-05

**Authors:** Jerome Aboab, Lisbet Niklason, Leif Uttman, Laurent Brochard, Björn Jonson

**Affiliations:** 1 Medical Intensive Care Unit, Hospital Henri Mondor, AP-HP, 51 Avenue du Marechal de Lattre de Tassigny, 94010 Créteil, France; 2 Department of Clinical Physiology, Lund University, University Hospital, 22185 Lund, Sweden; 3 Intensive Care Department, Geneva University Hospital, Rue Gabrielle-Perret-Gentil 4, 1205 Geneva, Switzerland

## Abstract

**Introduction:**

The inspiratory flow pattern influences CO_2 _elimination by affecting the time the tidal volume remains resident in alveoli. This time is expressed in terms of mean distribution time (MDT), which is the time available for distribution and diffusion of inspired tidal gas within resident alveolar gas. In healthy and sick pigs, abrupt cessation of inspiratory flow (that is, high end-inspiratory flow (EIF)), enhances CO_2 _elimination. The objective was to test the hypothesis that effects of inspiratory gas delivery pattern on CO_2 _exchange can be comprehensively described from the effects of MDT and EIF in patients with acute respiratory distress syndrome (ARDS).

**Methods:**

In a medical intensive care unit of a university hospital, ARDS patients were studied during sequences of breaths with varying inspiratory flow patterns. Patients were ventilated with a computer-controlled ventilator allowing single breaths to be modified with respect to durations of inspiratory flow and postinspiratory pause (T_P_), as well as the shape of the inspiratory flow wave. From the single-breath test for CO_2_, the volume of CO_2 _eliminated by each tidal breath was derived.

**Results:**

A long MDT, caused primarily by a long T_P_, led to importantly enhanced CO_2 _elimination. So did a high EIF. Effects of MDT and EIF were comprehensively described with a simple equation. Typically, an efficient and a less-efficient pattern of inspiration could result in ± 10% variation of CO_2 _elimination, and in individuals, up to 35%.

**Conclusions:**

In ARDS, CO_2 _elimination is importantly enhanced by an inspiratory flow pattern with long MDT and high EIF. An optimal inspiratory pattern allows a reduction of tidal volume and may be part of lung-protective ventilation.

## Introduction

Ventilator-induced lung injury is an important problem in the acute respiratory distress syndrome (ARDS). It may be caused by barotrauma related to high airway, alveolar, and transpulmonary pressures or by shear forces at lung collapse and opening during tidal breaths. Among efforts to provide lung-protective ventilation in ARDS, a reduction of tidal volume (V_T_) is a central issue [1-5]. By using lower than traditional V_T_, both of the mentioned damaging mechanisms may be mitigated. Recently, Bruhn *et al. *[6] showed by dynamic CT that cyclic collapse and opening is reduced by lower V_T_, providing direct evidence that low V_T _ventilation may be lung protective by reducing this phenomenon. However, the decrease in minute ventilation induced by low V_T _can be difficult to offset by increasing respiratory rate and may induce hypercapnia. So, adequate CO_2 _elimination under well-controlled airway pressure and tidal volume (V_T_) is an important clinical issue, particularly in ARDS.

**Figure 1:**
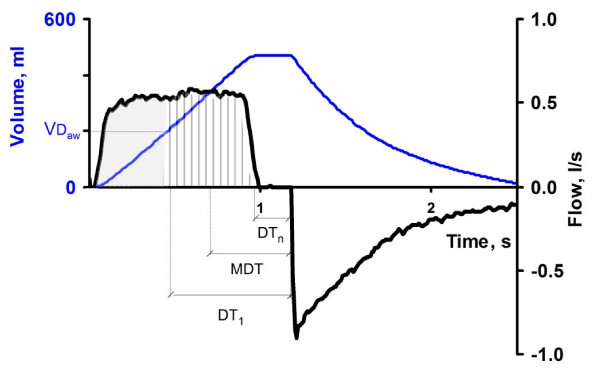
**Mean distribution time (MDT)**. MDT is the mean time during which consecutive fractions of inspired tidal volume remain in the respiratory zone of the lung (that is, the time available for distribution and mixing by diffusion of inspired gas with resident alveolar gas. The graph shows flow (black) and volume change (blue) of a breath against time. Until airway dead space (VDaw) has been inhaled (shaded area), no fresh gas arrives to alveoli, and this volume does not contribute to MDT. The following fractions of inhaled volume, N° 1 to N° n, (vertically striped area) have different distribution times in alveoli. For N° 1 distribution time is marked DT_1 _and for N° n DT_n_. MDT is the volume-weighted mean of DT_1 _to DT_n_.

**Figure 2:**
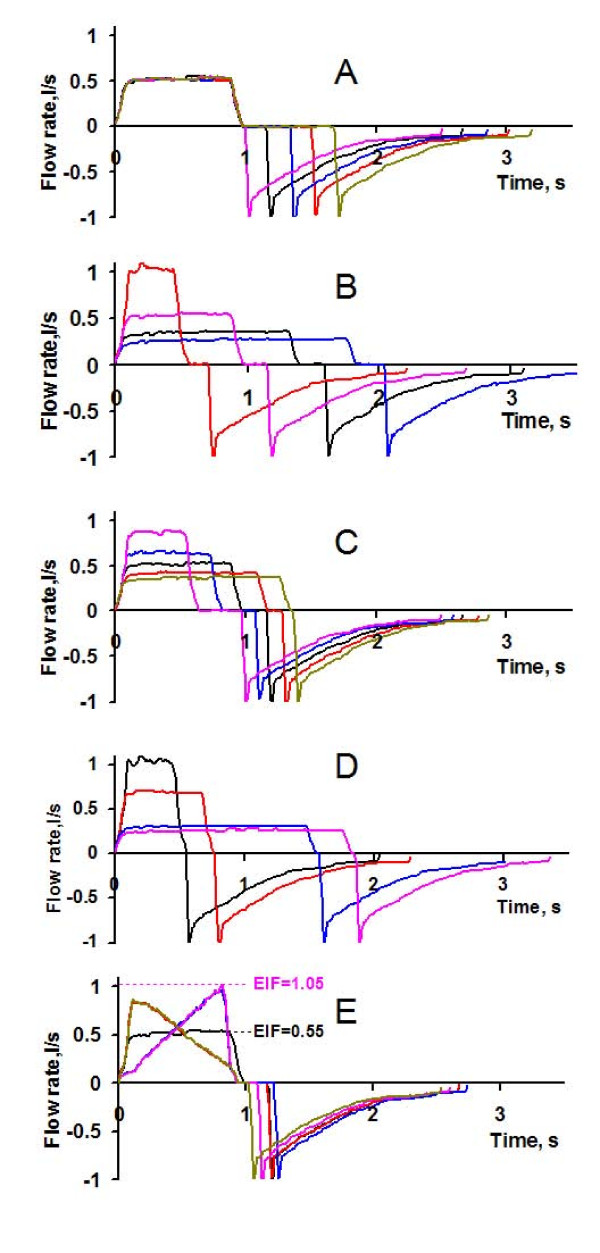
**Flow patterns**. Different flow patterns studied, all at similar V_T_, PEEP, and expiratory time. Inspiratory flow rate is positive. **(a) **Only T_P _modified. **(b) **Only T_I _modified. **(c) **T_I _and T_P _modified, maintaining constant MDT. **(d) **T_I _modified at shortest possible T_P _(1%). **(e) **Shape and T_P _modified. The definition of end-inspiratory flow (EIF) is illustrated for increasing flow and constant flow in **(e)**. For decreasing flow, EIF is zero, because flow rate ceases during inspiration.

Accordingly, dead-space reduction plays a role in a rational lung-protection strategy. This suggests an optimal pattern of inspiratory gas delivery [7-10]. Characteristics of this pattern are the time for gas insufflation (T_I_), the postinspiratory pause time (T_P_) after T_I_, and the inspiratory flow wave pattern, denoted SHAPE. SHAPE may be constant: that is, square flow, increasing; or accelerating flow or decreasing that is decelerating flow. T_I_, T_P_, and SHAPE affect mean distribution time (MDT), that is, time available for distribution and diffusion of inspired tidal gas with resident alveolar gas [6-8]. The definition of MDT is recapitulated in Figure 1. A longer T_P _prolongs MDT and enhances CO_2 _elimination [11]. It reduces dead space in healthy pigs and in pigs with acute lung injury [12]. The positive effects were more closely related to lnMDT than to MDT. In ARDS patients, a long T_P _reduces dead space and leads to reduced PaCO_2_, without a clinically significant increase in intrinsic positive end-expiratory pressure (PEEP) or negative hemodynamic effects [7,8]. In one study, the T_P _was varied, but also the T_I _and SHAPE. The results indicated that positive effects were related not only to a high value of MDT but also to an abrupt cessation of end-inspiratory flow (EIF) that follows from shortening T_I _or using an increasing flow pattern [12]. The definition of EIF is explained in Figure 2e.

No previous study investigated how various combinations of T_I_, T_P_, and SHAPE affect CO_2 _elimination in ARDS patients, and no previous study distinguished between effects of MDT and EIF. The objective of the present study was to test in ARDS patients the hypothesis that the effects of the inspiratory gas-delivery pattern on CO_2 _exchange can be comprehensively described from effects related to MDT, and also from effects of EIF, and to quantify the influence of these variables.

## Materials and methods

Eight mechanically ventilated subjects (Table 1) fulfilled criteria for ARDS [13]. Exclusion criteria were as follows: younger than 18 years, presence of a chest tube, contraindication to sedation or paralysis, intracranial disease, and a PaO_2_/F_I_O_2 _< 75 mm Hg. Sedation and neuromuscular blockade were achieved by continuous infusion of midazolam and atracurium. The Ethics Committee of French Intensive Care Society approved the protocol, which was part of another study looking at different PEEP settings and F_I_O_2 _[14]. This is the background behind the values of PEEP and F_I_O_2 _in individual patients. Patients' next of kin were informed and gave consent to the study and its publication.

**Table 1:** Characteristics of the subjects

Subject	Age	SAPS II	Cause of ARDS	Underlying disease	Days of ARDS
1	44	28	Sepsis	Meningitis	5
2	56	58	Septic shock	Lymphoma	3
3	68	73	Pneumonia	Thrombotic microangiopathy	2
4	58	64	Heat stroke	Alcoholism	0
5	66	81	Septic shock	Aortic valve replacement	5
6	64	76	Sepsis	Arterial hypertension	2
7	51	44	Pneumonia	Cirrhosis	0
8	75	73	Pneumonia	Obliterating artery disease	2

SAPS II, new simplified acute physiology score.

The patients were ventilated at volume control (ServoVentilator 900 C with a mainstream CO_2 _Analyzer 930; Siemens-Elema, Solna, Sweden). Each patient had an arterial line. For ordinary breaths during basal ventilation, T_I _was 33%, and T_P_, 10%. Set PEEP was 5 cm H_2_O. This low level of PEEP reflects the initial settings of the previous study [14]. The patients were ventilated with an effective V_T _of about 6 ml/kg ideal body weight [1]. Measured volumes were corrected with respect to gas compression in ventilator tubing. Individual ventilation parameters are given in Table 2.

**Table 2:** Ventilation characteristics of the subjects at baseline ventilation before measurements

Subject	**Effective V**_**T**_ (ml/kg IBW)	Respiratory rate (min^-1^)	Plateau pressure (cmH_2_O)	**F**_**I**_**O**_**2**_.	**PaO**_**2**_**/F**_**I**_**O**_**2**_ (mm Hg)	**PaCO**_**2**_ (mm Hg)
1	6.5	25	19	0.6	145	45
2	5.9	19	29	0.6	108	58
3	5.6	21	18	1.0	165	36
4	6.0	22	21	0.6	115	57
5	5.0	25	30	1.0	173	48
6	5.8	24	25	0.6	263	39
7	5.6	24	23	1.0	209	39
8	6.7	25	26	0.6	62	57

IBW, ideal body weight.

The ServoVentilator was controlled by a personal computer that emitted analog signals to the socket for external control of ventilator function [15,16]. By controlling respiratory rate and minute ventilation, the computer instantly controlled inspiratory flow rate and durations of inspiratory flow (T_I_) and postinspiratory pause (T_P_), while maintaining constant tidal volume [12].

The computer was programmed to perform six recording sequences, each comprising 12 consecutive breaths. Breaths numbers 3, 6, 9, and 12 were modified. In six different sequences, single breaths were modified with respect to T_I _(0.4 to 2.0 seconds), T_P _(0.0 to 0.9 seconds), and inspiratory flow wave form (SHAPE). In total, breaths with 20 different flow patterns were studied (Figure 2). As seen, SHAPE was square (constant flow) or triangular, with increasing or decreasing flow rate. Average values from the four ordinary breaths representing basal ventilation immediately preceding the modified breaths were used as reference for the modified breaths in each recording sequence. V_T_, PEEP, and expiratory time were constant for all breaths.

Signals representing airway flow and fraction of CO_2 _at airway opening in percentage (FCO_2_) were fed to the A/D converter of a personal computer and sampled at 100 Hz [12,16]. Recorded data were transferred to an Excel workbook for analysis (Microsoft Corp., Redmond, WA, USA).

**Figure 3:**
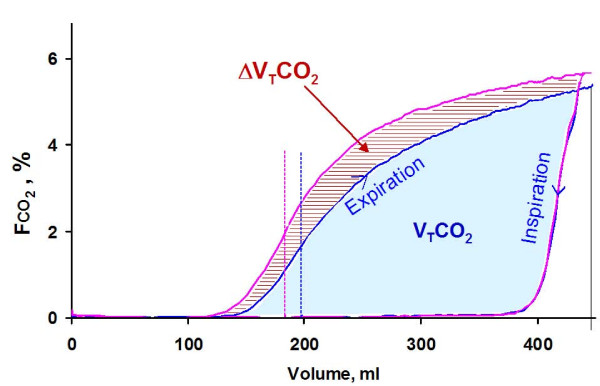
**Single-breath test for CO_2 _at ordinary and long postinspiratory pause in subject 4, depicting fraction of CO_2 _at airway opening, FCO_2_, against expired volume**. The blue loop shows the SBT-CO_2 _from an ordinary breath, and the magenta loop, a breath with a prolonged T_P_. The blue area corresponds to V_T_CO_2 _of an ordinary breath. The additional volume of CO_2 _eliminated at the longer T_P_, ΔV_T_CO_2_, indicated by hatched area, is caused partly by a lower-airway dead space (indicated by interrupted lines) and partly by a higher level of the alveolar plateau.

MDT was calculated from the average time that consecutive fractions of inspired gas remained in the respiratory zone, from their arrival in the zone until start of expiration (Figure 1) [7]. Further analysis was based on the single-breath test for CO_2_, SBT-CO_2_. SBT-CO_2 _allows calculation of airway dead space, essentially from the maximal rising slope of the curve, as described in detail by Åström *et al. *[17]. When PaCO_2 _is measured in steady state, alveolar dead space also can be determined [18]. Both dead-space fractions are affected by a modified inspiratory flow pattern, but only airway dead space can be studied at transient variations of ventilation. Therefore, in this study, the main studied variables were the volume of CO_2 _eliminated during a single tidal breath (V_T_CO_2_) and particularly the change in V_T_CO_2 _(ΔV_T_CO_2_) in breaths modified with respect to inspiratory flow pattern (Figure 3). ΔV_T_CO_2 _was expressed in percentage of V_T_CO_2 _of ordinary breaths in the same recording sequence (ΔV_T_CO_2_%). A positive ΔV_T_CO_2 _or ΔV_T_CO_2_% indicates enhanced CO_2 _elimination.

### Protocol

The subjects were studied in supine position when stable with respect to ventilation, blood pressure, heart rhythm, and metabolism, judged from CO_2 _elimination. If needed, endotracheal suction was performed well before the study and was not repeated during data collection, which lasted about 20 minutes. Each of the six types of recording sequences was in random order, performed twice, but in some patients, only once, when care of the patient was indicated.

### Statistical methods

Data are presented as mean ± standard deviation (SD). Regression analysis was used to study variations of volumes of CO_2 _in relation to parameters describing the inspiratory flow pattern. Significance implies that *P *< 0.05.

## Results

On average, 110 breaths were analyzed per patient, equal numbers of ordinary reference breaths and modified breaths with all combinations of T_I_, T_P_, and SHAPE (Figure 2). MDT varied between 0.12 seconds and 1.45 seconds, and EIF between 0 at decreasing flow and up to 1.7 L/sec, at increasing flow.

### T_P _variations: effects of MDT variation on ΔV_T_CO_2_%

When T_P _was increased, V_T_CO_2 _increased significantly because of lower-airway dead space and a higher alveolar plateau (Figure 3).

**Figure 4:**
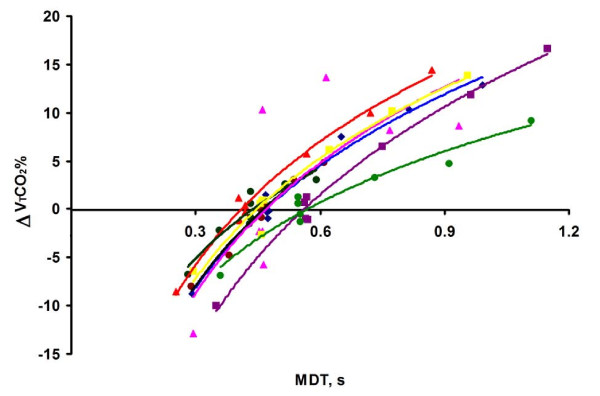
**Effects of T_P _variation**. When only T_P _was modified, ΔV_T_CO_2_% increased with MDT, shown by different colors for each patient. Each dot represents one breath. To illustrate the close correlation between ΔV_T_CO_2_% and lnMDT, lines for each subject represent the relation: ΔV_T_CO_2_% = *m *× lnMDT + *n*.

For all breaths in which only T_P _was modified, the ΔV_T_CO_2_% showed, in each patient, a strongly significant positive correlation to lnMDT; Figure 4. Notably, in these breaths, the inspiratory flow pattern was square, and V_T_, T_I_, and EIF were constant.

### Combined EIF and MDT variations: effects on ΔV_T_CO_2_%

**Figure 5:**
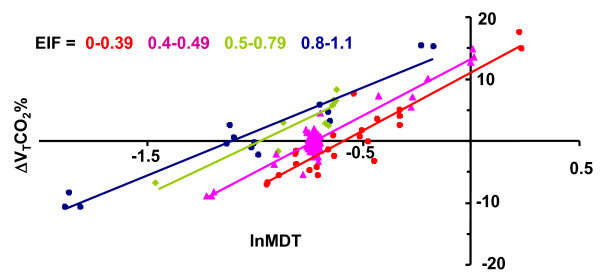
**ΔV_T_CO_2_% plotted against lnMDT in subject 4**. Groups of breaths with EIF within specified ranges are indicated by separate colors. For each range of EIF, a linear relation between lnMDT and ΔV_T_CO_2_% was observed.

In contrast to breaths modified only with respect to T_P_, in breaths modified with respect to SHAPE, no correlation was found between ΔV_T_CO_2_% and lnMDT. To explore the reason for this finding, all breaths of individual patients were separated into groups with narrow ranges of EIF and analyzed. Groups of breaths with high EIF were at specific values of MDT associated with high ΔV_T_CO_2% _(Figure 5). Within each group of breaths with similar EIF, the ΔV_T_CO_2_% strongly correlated to lnMDT.

The ΔV_T_CO_2_% was, for all breaths in each subject, correlated to lnMDT and EIF by using the equation:

(1) $$\Delta {\text{V}}_{\text{T}}\text{C}{\text{O}}_{\text{2}}\%=a\times \text{ln}\text{MDT}+b\times \text{EIF}+c$$

To translate data of coefficients *a, b *and *c *(Table 3) to information more comprehensible from a clinical point of view, the ΔV_T_CO_2_% was calculated for different inspiratory flow patterns (Table 4). The calculations were based on mean values among subjects for coefficients *a, b *and *c *in Eq. 1 and values for the subject with the highest coefficient *b *that indicates a strong influence of EIF (subject 4).

**Table 3:** Values of *a, b*, and *c *in each subject according to the equation: $\Delta {\text{V}}_{\text{T}}\text{C}{\text{O}}_{\text{2}}\%=a\times \text{ln}\text{MDT}+b\times \text{EIF}+c$

Subject	*a*	*b*	*c*	*R^2^*
1	12.9	2.3	9.0	0.71
2	16.4	11.5	4.8	0.76
3	11.7	6.4	4.6	0.59
4	16.7	12.8	6.4	0.84
5	14.5	0.4^NS^	12.9	0.74
6	13.4	5.6	7.8	0.34
7	15.6	1.9^NS^	11.0	0.58
8	10.9	-2.2^NS^	10.4	0.40
**Mean**	**14.0**	**4.8**	**8.4**	**0.62**
**SD**	**2.2**	**5.3**	**3.0**	**0.18**

NS, not significantly different from zero.

**Table 4:** Effects of different inspiratory patterns on EIF, MDT, and lnMDT

	T_I _33%, T_P _10%			T_I _15%, T_P _28%			T_I _15%, T_P _35%		
SHAPE	Increasing flow	Constant flow	Decreasing flow	Increasing flow	Constant flow	Decreasing flow	Increasing flow	Constant flow	Decreasing flow
EIF, ml/s	1,106	553	0	2,433	1,217	0	2,433	1,217	0
MDT	0.41	0.52	0.71	0.80	0.86	0.95	0.99	1.05	1.14
lnMDT	-0.90	-0.66	-0.34	-0.22	-0.15	-0.05	-0.01	0.05	0.13
ΔV_T_CO_2_%, all subjects	1	2	4	17	12	8	20	15	10
ΔV_T_CO_2_%, subject 4	5	2	1	34	19	6	37	23	9

Effects on ΔV_T_CO_2 _were calculated by using mean coefficients *a *and *b *for all subjects (Eq. 1) and coefficients for subject 4 who had a high coefficient *b*.

At T_I _33% and T_P _10%, one observes that for mean *a, b *and c values, a decreasing flow has a slightly positive ΔV_T_CO_2_% (Table 4, left columns). This reflects that, in most patients, the positive effect of a longer MDT outweighs a modest negative effect of an EIF that is zero at decreasing flow. In contrast, in subject 4, increasing flow implying a high EIF enhanced CO_2 _elimination.

If one would redistribute the total inspiratory time by shortening T_I _and prolonging T_P_, the ΔV_T_CO_2_% would increase, and much more so at increasing flow because of high EIF at that pattern (Table 4, middle columns). The effect would be very important in subject 4. Further enhancement of CO_2 _elimination would result from an increase in total inspiratory time from 43% to 50% of the respiratory cycle (Table 4, right columns).

## Discussion

The computer-controlled ventilator allowed elaborate modification of inspiratory flow wave pattern on a breath-by-breath basis at constant tidal volume. Within 20 minutes of study time, about 20 types of modified breaths were studied. The ΔV_T_CO_2_% expresses how an alternative inspiratory pattern affects V_T_CO_2 _relative to ordinary breaths in the same recording sequence. Thereby, changes in CO_2 _exchange due to variable physiological conditions between patients and in a single patient with time were minimized. With this technique and focusing on ΔV_T_CO_2_, we could for the first time comprehensively describe and distinguish effects of MDT and EIF in ARDS patients.

The most uncomplicated breath modification is when only T_P _was modified, as this does not affect EIF. The findings confirm that the ΔV_T_CO_2_% is significantly affected by the length of the pause and tightly positively correlated to lnMDT [7,12]. A comprehensive analysis of variation in T_I_, T_P_, and SHAPE has previously not been performed in patients. Such analysis showed that both MDT and EIF influence the V_T_CO_2 _and that the effects can be described with the simple equation:

(2) $$\Delta {\text{V}}_{\text{T}}\text{C}{\text{O}}_{\text{2}}\%=a\cdot \text{ln}\text{MDT}+b\cdot \text{EIF}+c$$

At decreasing flow, more inhaled gas reaches alveoli early during inspiration. Thereby, MDT is prolonged. This will tend to enhance CO_2 _exchange. However, EIF and MDT are negatively correlated to one another for breaths varied with respect to SHAPE. For example, for breaths with decreasing flow, MDT is long, but EIF is zero. This implies that EIF will balance and obscure effects of MDT; in agreement with that, no correlation between ΔV_T_CO_2_% and MDT was found among breaths with varying SHAPE.

Gas transfer over the boundary zone between fresh inhaled gas and resident alveolar gas and gas mixing within the alveolar zone are complex phenomena. Diffusion is a strictly time-dependent phenomenon and is believed to be the main phenomenon behind effects of MDT in ARDS. Diffusion drives gas transfer through the whole respiratory zone between capillary blood and conducting airways. In 1970, Knelson *et al. *[12] reported that a postinspiratory pause led to a more efficient alveolar gas exchange. They showed theoretically that this is due not only to improved distribution of ventilation but also to alveolar perfusion during the pause during which alveolar CO_2 _tension approaches that of mixed venous blood. Fletcher *et al. *[19] further developed this concept. Aboab *et al. *[14] reasoned that a higher level of the alveolar plateau associated with a long pause might partly be due to continuing delivery of CO_2 _by alveolar perfusion. In their study, a detailed analysis of breaths with similar MDT but variable distribution between T_I _and T_P _led to the suggestion that time for alveolar perfusion during inspiration is of low importance compared with time for distribution and diffusion within the alveolar zone, as expressed by MDT. Still, delivery of CO_2 _to alveoli during T_I _and T_P _may to some extent contribute to positive effects of decreasing flow and a long T_P_.

In a theoretic study, Jansson and Jonson [20] showed that a decreasing flow in combination with postinspiratory pause is favorable with respect to an even distribution of ventilation in the presence of uneven airway obstruction, a phenomenon associated with so-called *pendelluft*. They also postulated a small opposite effect in lungs with uneven compliance. In ARDS, effects on gas distribution to different lung regions are complex. Nevertheless, the influence of the inspiratory pattern is thought to relate more closely to diffusion within lung units than to distribution of ventilation between lung units.

Effects of EIF may reflect that high-frequency flow and pressure transients at airway opening are transmitted through the airways to the periphery of the lungs. This is the basis behind impulse oscillometry used for diagnostic purposes [21,22]. In mechanical ventilation, it also is important for high-frequency oscillation [23]. Sudden interruption of flow at the end of inspiration, as expressed by EIF, implies that oscillations covering a broad spectrum of frequencies are transmitted through the airways down to the alveolar zone. Such oscillations serve to mix gas in the boundary zone between conducting airways and alveoli. This is the conceivable mechanism for the observed positive effect of a high EIF on CO_2 _exchange.

The effect of the MDT on the ΔV_T_CO_2_% was similar among subjects (Figure 4, Table 3). In contrast, the effect of EIF varied importantly among subjects, as shown by large variation in coefficient *b*. The basis behind impulse oscillometry is that transmission of flow oscillations through the airways reflects differences in distributed resistance, compliance, and inertia along the airways. As lung mechanics is complexly perturbed in ARDS, the finding that EIF is of variable importance among patients could be expected.

In the present study, CO_2 _exchange was studied only for single modified breaths at a time. It has been shown that a change in V_T_CO_2 _measured with the present technique will be followed by a corresponding change in PaCO_2 _in the opposite direction [8,10,12,24]. At constant respiratory rate, an observed change in V_T_CO_2 _after a change in tidal volume or inspiratory flow pattern will affect PaCO_2 _in animals and in humans, in health and in disease. The layout of this study, based on studies of single breaths, has the strength of making it possible to study a large spectrum of inspiration and pause patterns within a short period, during which the physiological status of the patient remains essentially stable. Conversely, further studies are indicated in which particular patterns of inspiration are studied in steady state to evaluate effects on arterial blood gases, physiological dead space, and other parameters.

The results show that it is possible to enhance CO_2 _elimination by about 12% to 15% just by modifying T_I _and T_P _at constant V_T _in most ARDS patients and to about 20% in some patients (Table 4). At long T_P_, further enhancement is possible by using increasing inspiratory flow. On modification of T_I _and T_P _leading to a higher I:E ratio, we could until now only partly understand clinical observations of immediate very important increments in CO_2 _elimination observed on the 930 CO_2 _Analyzer, followed by corresponding decrease in PaCO_2 _after a switch to a more efficient pattern of inspiration. On the basis of the present results, an I:E ratio of 1:1 with a long T_P _seems appropriate.

In most ARDS patients, it is more important to reduce V_T _than to enhance CO_2 _elimination. This would be the case in the present material in which hypercapnia was not a problem. Hypercapnia may have a lung-protective effect in itself. Such effects are not proven to improve outcome in ARDS [25]. However, hypercapnia combined with an efficient pattern of inspiration will allow particularly low tidal volumes, which may enhance lung protection. A combination of methods and approaches is needed for optimal reduction of dead space. Jonson *et al. *[26] showed that expiratory flushing of airways, later denoted tracheal gas injection [27], may be used to clear the airways from CO_2 _down to trachea. To avoid potential problems of humidification of injected gas and of jet streams in the trachea, aspiration of dead space (ASPIDS) was developed and tested in animals and patients [28,29]. With ASPIDS, dead-space gas is aspirated during late expiration through a catheter at the tip of the tracheal tube and simultaneously replaced by an equally large flow of fresh gas through the ordinary inspiratory pathway, avoiding all other influences on ventilation. In a porcine ARDS model, one can, with a combination of ASPIDS and the MDT concept, achieve normocapnia at very low V_T _ventilation, as shown by Uttman *et al. *[10]. Despite the high metabolic rate in adolescent pigs, V_T _was 4 ml/kg body weight. Notably, this very low V_T _was achieved at respiratory rates of about 80 breaths/min. At high respiratory rates, MDT becomes shorter. Then, it is particularly important to choose a ventilation pattern that is optimal with respect to CO_2 _exchange, which is a short T_I_, a long T_P_, combined with a short expiratory phase. This may augment intrinsic PEEP, an effect that should be balanced by a reduction of set PEEP. The study of Uttman *et al. *[10] illustrates that a reduced dead space paves the way for a higher respiratory rate. Theoretically, at dead space approaching zero, V_T _could be reduced toward zero at very high respiratory rates.

Right-to-left intrapulmonary shunt contributes to alveolar dead space. As recently shown, this contribution increases at high metabolic rate, low cardiac output, low hemoglobin concentration, metabolic acidosis, and respiratory alkalosis [30]. Accordingly, conventional critical care measures aiming at homeostasis have the advantage of dead-space reduction. By addressing all **means**for dead-space reduction, in combination with much higher respiratory rates and much lower tidal volume than conventionally applied, adequate CO_2 _elimination may be achieved in ARDS patients. Eventually one may reduce the use of extracorporeal gas exchange. The field for research remains wide open

## Conclusions

CO_2 _exchange at different inspiratory patterns can be described according to a simple equation based on MDT and EIF. Just by setting the ventilator to a pattern that enhances CO_2 _exchange, one may reduce dead space and significantly increase CO_2 _elimination or alternatively reduce V_T_. This option merits use in clinical routine and, particularly, in further studies of optimal ventilation in ARDS.

## Key messages

• In ARDS, CO_2 _exchange is importantly affected by the inspiratory flow wave pattern.

• Mean distribution time (MDT) and end-inspiratory flow (EIF) influence of CO_2 _exchange, as expressed with a simple equation.

• The effect of MDT is similar among ARDS patients, whereas that of EIF is variable.

• A short insufflation followed by a long postinspiratory pause enhances CO_2 _exchange.

• An efficient pattern of insufflation may be lung protective by allowing a lower tidal volume.

## Abbreviations

ARDS: acute respiratory distress syndrome; EIF: end-inspiratory flow; FCO_2_: fraction of CO_2 _at airway opening; F_I_O_2_: fractional inspired oxygen; IBW: ideal body weight; MDT: mean distribution time; PaCO_2_: arterial carbon dioxide tension; PaO_2_: arterial oxygen tension; PEEP: positive end-expiratory pressure; SAPS II: new simplified acute physiology score; SBT-CO_2_: single-breath test for CO_2_; T_I_: insufflation time; T_P_: postinspiratory pause time; V_T_: tidal volume; V_T_CO_2_: eliminated volume of CO_2 _per breath; ΔV_T_CO_2_%: change in V_T_CO_2 _in percentage of the value of ordinary breaths.

## Competing interests

The authors declare that they have no competing interests.

## Authors' contributions

BJ, LB, JA, and LU participated in study design. JA and BJ performed data collection. LN made computer programs and performed primary data analysis. All authors participated in manuscript preparation and read and approved the final manuscript.
